# From frozen cell bank to product assay: high-throughput strain characterisation for autonomous Design-Build-Test-Learn cycles

**DOI:** 10.1186/s12934-023-02140-z

**Published:** 2023-07-14

**Authors:** Laura M. Helleckes, Debora Puchta, Hannah Czech, Holger Morschett, Bertram Geinitz, Wolfgang Wiechert, Marco Oldiges

**Affiliations:** 1 Institute for Bio- and Geosciences: IBG-1, Forschungszentrum Jülich, Jülich, Germany; 2 Institute of Biotechnology, RWTH Aachen University, Aachen, Germany; 3 Computational Systems Biotechnology, RWTH Aachen University, Aachen, Germany

**Keywords:** Bioprocess development, Automation, Microbioreactor, Clean-in-place, Design-Build-Test-Learn

## Abstract

**Background:**

Modern genome editing enables rapid construction of genetic variants, which are further developed in Design-Build-Test-Learn cycles. To operate such cycles in high throughput, fully automated screening, including cultivation and analytics, is crucial in the Test phase. Here, we present the required steps to meet these demands, resulting in an automated microbioreactor platform that facilitates autonomous phenotyping from cryo culture to product assay.

**Results:**

First, an automated deep freezer was integrated into the robotic platform to provide working cell banks at all times. A mobile cart allows flexible docking of the freezer to multiple platforms. Next, precultures were integrated within the microtiter plate for cultivation, resulting in highly reproducible main cultures as demonstrated for *Corynebacterium glutamicum*. To avoid manual exchange of microtiter plates after cultivation, two clean-in-place strategies were established and validated, resulting in restored sterile conditions within two hours. Combined with the previous steps, these changes enable a flexible start of experiments and greatly increase the walk-away time.

**Conclusions:**

Overall, this work demonstrates the capability of our microbioreactor platform to perform autonomous, consecutive cultivation and phenotyping experiments. As highlighted in a case study of cutinase-secreting strains of *C. glutamicum*, the new procedure allows for flexible experimentation without human interaction while maintaining high reproducibility in early-stage screening processes.

**Supplementary Information:**

The online version contains supplementary material available at 10.1186/s12934-023-02140-z.

## Background

Modern industrial biotechnology relies on a complex interplay of genetically engineered microbial cell factories and optimised process conditions. Recent advances in genome editing technology allow for fast generation of thousands of genetically engineered strain variants in short time. In biofoundries, these are subject to Design-Build-Test-Learn (DBTL) cycles to develop production strains on short time scales [1]. While large-scale stirred tank reactors are mostly used in production processes, early screening of such libraries is often performed in small-scale shaken bioreactors [2], where microbioreactors (MBRs) are of particular interest [3].

Most notably, a high degree of parallelisation and small culture volumes result in greatly increased throughput, thereby supporting fast and cost-efficient process development. In recent years, MBR systems have been developed both based on miniaturised stirred tank reactors (e.g. [4]) or microtiter plates (MTPs) (e.g. [5, 6]). However, several limitations remain with stand-alone MBR systems. Most prominently, product analysis often requires manual sampling for offline measurements. When shaking is paused, however, sampling may interfere with the cultivation [7]. Here, automation, more specifically the combination of MBRs with liquid handling robotic platforms, was shown to be beneficial, e.g. for non-invasive, high-throughput (HT) sampling [8].

**Fig. 1:**
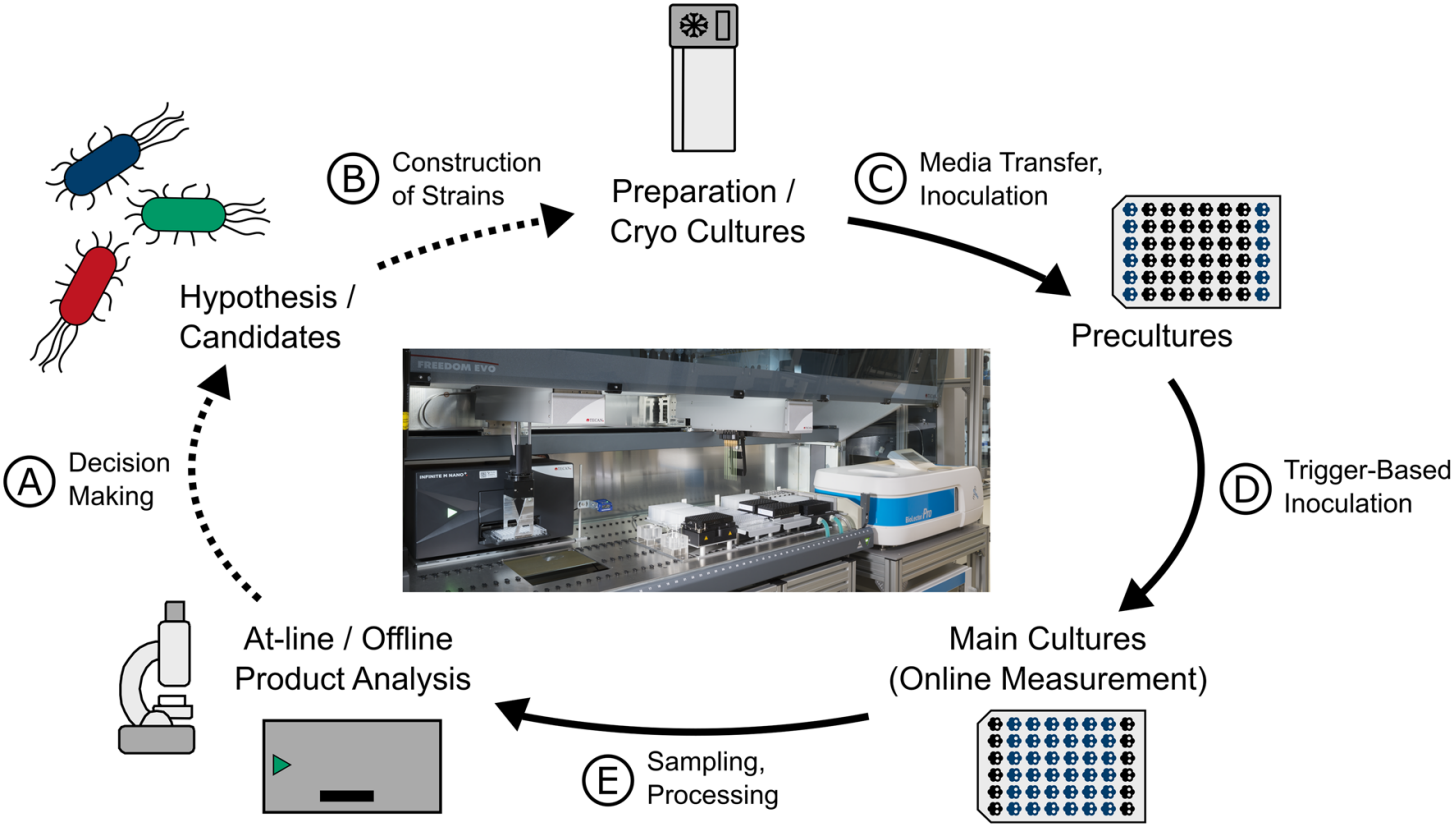
Steps of autonomous strain library screening as part of DBTL. This representation sets the focus on experimental steps required for strain library screening, which is the Test phase of DBTL. As for the other steps of DBTL, indicated by the dashed lines, the Design and Learn phases are summarised in task $\textcircled {A}$ while the Build step is represented in task $\textcircled {B}$. To demonstrate the experimental capabilities for autonomous DBTL, tasks $\textcircled {A}$ and $\textcircled {B}$ can be replaced by the screening of pre-defined strain designs

Several examples for such platforms exist [9–11, 13]. In previous studies, a platform combining the commercial BioLector^®^, an MTP-based system, with robotic workflows and photometric assays was applied for advanced protocols such as enzyme ﻿﻿﻿scre﻿eni﻿ng﻿﻿ [12, 13], detection of production kinetics and substrate uptake [7] as well as adaptive laboratory evolution [14]. Building upon these studies, fully autonomous screening procedures for thousands of variants can be targeted, which are required to drive the full DBTL cycle. The experimental steps required for such a screening using a BioLector^®^ Pro as MBR are shown in Fig. 1.

In this work, we focus on the Test phase of DBTL and its necessary steps. The first two phases of the cycle, Design and Build, are thus summarised as task $\textcircled {A}$ and task $\textcircled {B}$ and not further detailed. Regarding the Test phase, each screening experiment begins with the provision of working cell banks (WCBs), where strains are mostly stored via cryopreservation in case of bacteria or cell lines (Fig. 1 top). In subsequent task $\textcircled {C}$, precultures, which are used to increase cell fitness and reproducibility [15], are prepared from cultivation medium and a WCB. Main cultures are then inoculated from these precultures in task $\textcircled {D}$ and subsequently monitored via quasi-continuous online measurements. In case of a BioLector^®^ Pro system, biomass, pH and dissolved oxygen as well as fluorescence can be measured. For further analysis of process parameters, sampling (task $\textcircled {E}$) and subsequent at-line or offline analytics can be required [16], for example via repetitive, low-volume sampling [17]. Finally, data analysis and modelling are instrumental in determining key performance indicators based on which the strains and conditions can be ranked. In the process of decision making, which includes the Learn phase of DBTL, new variants or experimental designs are then suggested for the next round of screening, thereby closing the cycle.

Operation of the cycle with many autonomous repetitions is necessary to meet the speed of HT strain generation in task $\textcircled {B}$. Besides the digital infrastructure that is essential for seamless operation of consecutive steps, e.g. process control and data management systems, several technical requirements so far limit this vision, resulting in partial automation as the status quo in academic context. First, cryo cultures are mostly provided manually for inoculation by the robotic liquid handler [13, 18] or alternatively, preculture medium is manually inoculated under sterile conditions prior to the automated experiment [16]. Both approaches reduce flexibility of process initialisation since they strictly require human interaction, thus preventing autonomous cycles. Even in a study where a deep freezer was available on the robotic platform, it was only used for sample storage [13]. This could be explained by the lack of small, automated freezers that enable temperatures lower than −20 $^\circ$C, avoiding temperatures that are unusually high for bacterial cryopreservation [19]. As a second challenge, precultures are often performed externally in shake flasks or in a separate BioLector^®^ run prior to the robotic experiment (e.g. [17]). Here, recent studies  [12, 16, 20] started to use dedicated wells of the cultivation MTP for precultures, thus saving time and entering the automated process earlier. Finally, even if WCBs and precultures are handled by robotic procedures, a remaining challenge for autonomous DBTL is the availability of a sterile cultivation MTP in the BioLector^®^ for the start of the next cultivation run, which needs to be manually put in place by a human operator.

In this study, we present a set of technically and experimentally required steps to overcome these challenges. We demonstrate how integration of an automated deep freezer allows flexible starts of experiments without the presence of a human operator. Combined with integrated precultures and media handling, all necessary steps for preparation of main cultures can be automated. Furthermore, we introduce two clean-in-place (CIP) procedures to restore sterile conditions in cultivation MTPs, thus allowing for consecutive batch cultivations without human interaction. In a final case study with cutinase-secreting *Corynebacterium glutamicum* strains, we bring this methodology to practice and highlight the advantages of autonomous, consecutive screening rounds for statistically meaningful experimental designs, thus enabling an autonomous Test phase within DBTL.

## Results and discussion

### Step 1: integration of automated deep freezer

The autonomous handling of WCBs during screening experiments requires the integration of a deep freezer into the existing robotic platform. As described in section "Integration of automatic deep freezer", the selected, small-sized automated freezer (LiCONiC, Liechtenstein) was attached to the liquid handling robot via a cut-out in the front window, which can be sealed when the freezer is not needed for experiments. The designed mobile cart (Additional file 1: Fig. S6) allows usage on multiple liquid handling units, which is beneficial for laboratories with varying users and experimental layouts.

Deep freezers for cryopreserved cells are usually operated at temperatures between −70 $^\circ$C and −80 $^\circ$C to prevent reduction of viable cell count and ensure reproducible growth after thawing [19]. Before the deep freezer was used in experimental application, the cell viability thus needed to be verified over the expected storage duration of maximum six weeks at −20 $^\circ$C, which is the lowest temperature the freezer can be operated at. For this, cryo cultures of *C. glutamicum* were manually produced as described in section "Cell viability studies with *C. glutamicum*" and stored at −80 $^\circ$C and −20 $^\circ$C, respectively. As a measure for reproducibility, the batch times were calculated from 12 replicates as explained in section "Cell viability studies with *C. glutamicum*". The results are shown in Table 1.

**Table 1:** Batch times of cultures inoculated from cryo cultures stored at −80 $^\circ$C and −20 $^\circ$C

Week	Batch time (−80 $^\circ$C) [h]	Batch time (−20 $^\circ$C) [h]
0	$12.73 \pm 0.06$	$12.80 \pm 0.11$
1	$12.51 \pm 0.07$	$13.51 \pm 0.23$
2	$12.86 \pm 0.07$	$13.64 \pm 0.24$
3	$12.77 \pm 0.07$	$13.93 \pm 0.07$
4	$12.94 \pm 0.07$	$13.97 \pm 0.11$
5	$12.99 \pm 0.09$	$14.23 \pm 0.07$
6	$12.78 \pm 0.07$	$13.95 \pm 0.09$

Batch times were investigated weekly over the course of six weeks by cultivation and subsequent spline analysis of the growth curves (section Spline analysis for batch time calculcation). For each of the storage conditions and weeks, three different cryo cultures were used, each of those for inoculation of four wells to an optical density (OD) of 0.1. This leads to a number of 12 replicates per storage condition and week. A minimum error in time of 4 min was assumed since this is the cycle time used for measurements in the BioLector^®^

First, it can be seen that cultures inoculated at week 0, right after production of WCBs, show no difference between −20 $^\circ$C and −80 $^\circ$C and thus serve as a reference for the following weeks. After a storage period of one week, it became evident that the higher temperature of −20 $^\circ$C led to an increased batch time of about 1 h (8%) compared to –80 $^\circ$C. This effect continues after week 1. However, the largest increase in batch times between weeks was observed from week 0 to 1 for −20 $^\circ$C, while the average batch times over the course of the remaining 5 weeks stay within a difference of less than 1 h to each other. This indicates that the higher storage temperature has an initial influence on the cell viability, but the effect for long-term storage over six weeks is rather small. In another comparison, the differences between the average batch times of the two temperatures per week only vary between 0.78 h (week 2) and 1.24 h (week 5) after week 1, so that storage at −20 $^\circ$C is acceptable for six weeks. It can also be observed that reproducibility between the 12 culture replicates (three biological replicates) per week remains high throughout the study with < 2% relative deviation in the calculated batch times for both conditions. Longer storage times are likely to lead to comparable results, but were not further investigated since a period of six weeks is sufficient to perform screening experiments for a variant library on the robotic platform. While *C. glutamicum* was chosen for the final case study and the respective data is thus discussed here, storage at −20 $^\circ$C was also validated with *Escherichia coli*, leading to similar results (Additional File 1, Table S1).

**Fig. 2:**
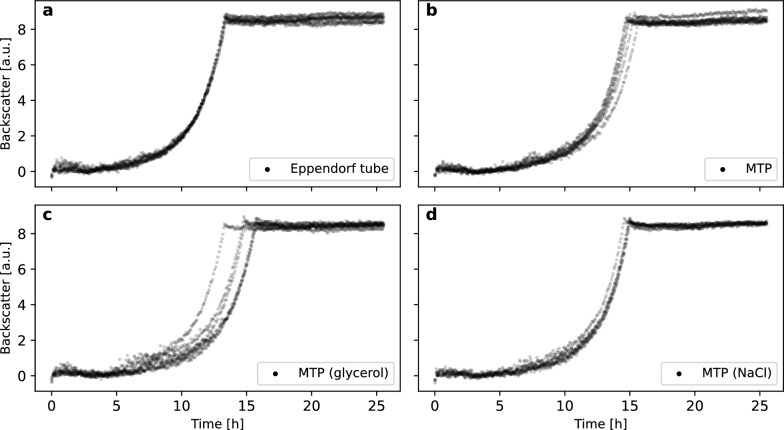
Comparison of preculture reproducibility after inoculation from different cryo cultures. **a** Storage in Eppendorf Tubes^®^ with OD 20, glycerol content of 25% (w/v), manual dilution to OD 4 with 0.9% (w/v) NaCl solution before inoculation. **b** Storage in MTP with OD 4, glycerol content of 25% (w/v), directly used for inoculation. **c** Storage in MTP with OD 8, glycerol content of 25% (w/v), addition of pre-warmed 50% (w/v) glycerol by liquid handler before inoculation. **d**: Storage in MTP with OD 8, glycerol content of 25% (w/v), addition of pre-warmed 0.9% (w/v) NaCl solution by liquid handler before inoculation

For autonomous cultivation workflows with the deep freezer, inoculation from WCBs stored in MTPs is required. The status quo from previous studies on the robotic platform are cryo cultures in cryo vials or Eppendorf Tubes^®^ (e.g. [12]) that are manually thawed and diluted. For comparison to the new MTP-based strategy, cryo cultures in Eppendorf Tubes^®^ (section "Inoculation tests with MTPs") were thawed and diluted to an OD of 4 with sterile 0.9% (w/v) NaCl solution. While this procedure is not well suited for autonomous handling, it leads to high reproducibility in the microbioreactor experiments, as shown in Fig. 2a, and thus serves as a benchmark.

For comparison, the WCBs in MTPs at OD 4 were used for inoculation after 7 min thawing time (Fig. 2b). Here, a higher variance in batch times was observed compared to the standard procedure with Eppendorf Tubes^®^. One difference between the two approaches is the different content of glycerol. Since the MTP-based WCBs were adapted to OD 4 before freezing, they have a higher glycerol content of 25%. The Eppendorf Tubes^®^, however, were frozen at OD 20 and subsequently diluted to OD 4, thus containing only 5% glycerol. Since the liquid handler was operated with settings for water-like fluids in both cases, inaccurate pipetting is likely to explain the higher variance for the MTPs. To further investigate this hypothesis, WCBs in MTPs were used in two other approaches. In one case, 100 $\upmu$L NaCl solution at 40 $^\circ$C was added to an MTP as described in section "Inoculation tests with MTPs". In the second case, 100 $\upmu$L pre-warmed 50% (w/v) glycerol was added. The results can be seen in Fig. 2d and c respectively.

Notably, the addition of NaCl improved the reproducibility while the addition of glycerol led to high standard deviations in batch times. The addition of NaCl lowers the glycerol content per well to 12.5%, thus creating more water-like conditions that are easier to pipet. While it would have been possible to adapt the liquid handling settings to higher glycerol amounts, the addition of NaCl at 40 $^\circ$C reduced the thawing time from 7 min to about 45 s, thus shortening the overall process time significantly. In the following, these conditions were thus chosen for inoculation. Having implemented a suitable inoculation strategy, the next step is to integrate precultures into the robotic workflows.

### Step 2: integrated preculture strategy for high reproducibility

**Fig. 3:**
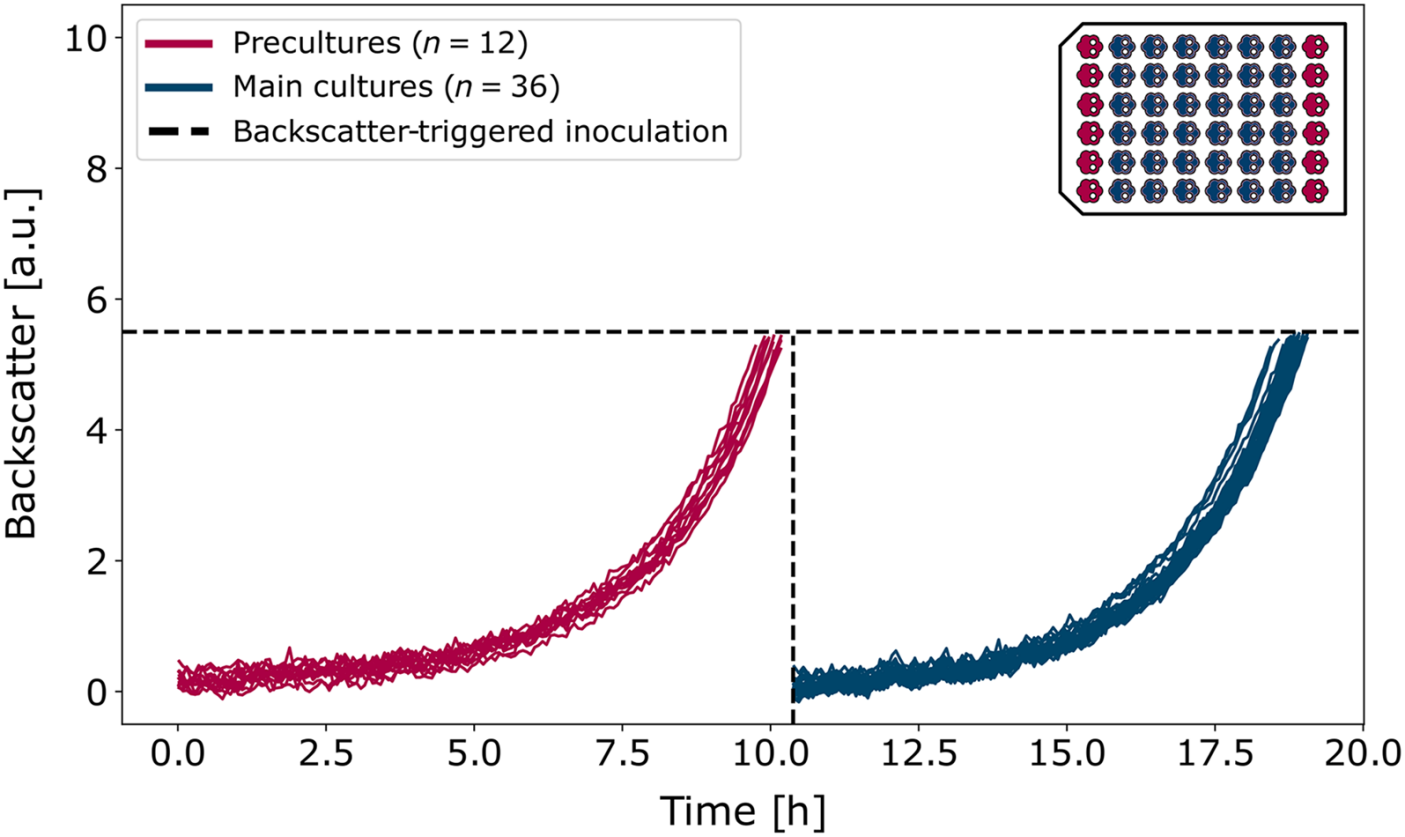
Pre- and main cultures conducted within the same FlowerPlate^®^. 12 precultures (red) of *C. glutamicum* were inoculated in the outer columns of the cultivation MTP as shown in the inlay plot. Upon a backscatter trigger of 5.5 a.u., three times 20 $\upmu$L of each preculture was used to inoculate the respective main cultures (blue). The 36 main cultures were individually sampled at a backscatter of 5.5 a.u

For full automation of the described process, conventional approaches for small-scale precultures such as test-tube or shake-flask overnight cultures are not suitable since they cannot be handled on the robotic platform. Accordingly, precultures were to be integrated into the BioLector^®^ cultivation process. Since different layouts of splitting wells among pre- and main cultures can be chosen, depending on the organism and purpose of the experiment, the experimental control script (section "Robotic platform for HT cultivation and screening") was programmed to support different layouts. Here, we present results for 12 precultures being inoculated in the outer columns of the cultivation MTP and for the corresponding triplicate design of main cultures in the adjacent inner wells (Fig. 3, top right inlay). In all following experiments, FlowerPlates^®^ served as cultivation MTPs.

All 12 precultures grow with high reproducibility as shown in Fig. 3 (red curves). Upon an individual backscatter trigger of 5.5 a.u., which was reached after a batch time of 10.12 h ± 0.14 h (Additional file 1: Table S3) as indicated by the dashed lines, three times 20 $\upmu$L of each preculture were used to inoculate main cultures, resulting in 36 cultures shown in blue. These main cultures were sampled at a backscatter of 5.5 a.u. individually to resemble a procedure with offline analytics. Again, the high reproducibility of growth triplicates (batch time 8.48 h ± 0.10 h, Additional file 1: Table S3) indicates a successful implementation of automated precultures for *C. glutamicum*. The final step thus is the CIP procedure to allow for autonomous, consecutive cultivation.

### Step 3: CIP strategies for cultivation MTP

When applying CIP strategies for cultivation MTPs, three major objectives need to be addressed: sterility, consistent process conditions and little attrition of the materials in use. To ensure sterile conditions, all CIP strategies used in this paper include an incubation step with a disinfectant whose composition is similar to commercially available solutions (section "Clean-in-place procedure"). To secure unchanged process conditions for the next cultivation round, this disinfectant has to be removed after incubation. Such removal was intended to be handled under shaken conditions for the MTP, which is required to keep the process flexible for different cultivation times of different wells, i.e., to allow removal while other wells are still in cultivation mode. Due to limitations of robotic needle positioning during shaking, small amounts of residual liquid (around 10 $\upmu$L) remain in the well even after optimisation of the liquid handling parameters (section "Clean-in-place procedure"). These residuals need to be removed by methods other than pipetting. Two such strategies were tested and critically evaluated as described in section "Clean-in-place procedure": evaporation or dilution with medium. The first one includes usage of methanol as a solvent, which could impede the adhesion of MTP parts, especially the transparent bottom plate. As a potential benefit, methanol and its metabolites are cytotoxic to *C. glutamicum* [21] and many other microbial systems [22], acting as a second disinfectant. For the second strategy with medium, the ability to restore sterile and consistent process conditions needed to be validated. In the following, results for both strategies are shown and discussed in detail.

#### CIP strategy with methanol for disinfectant removal

**Fig. 4:**
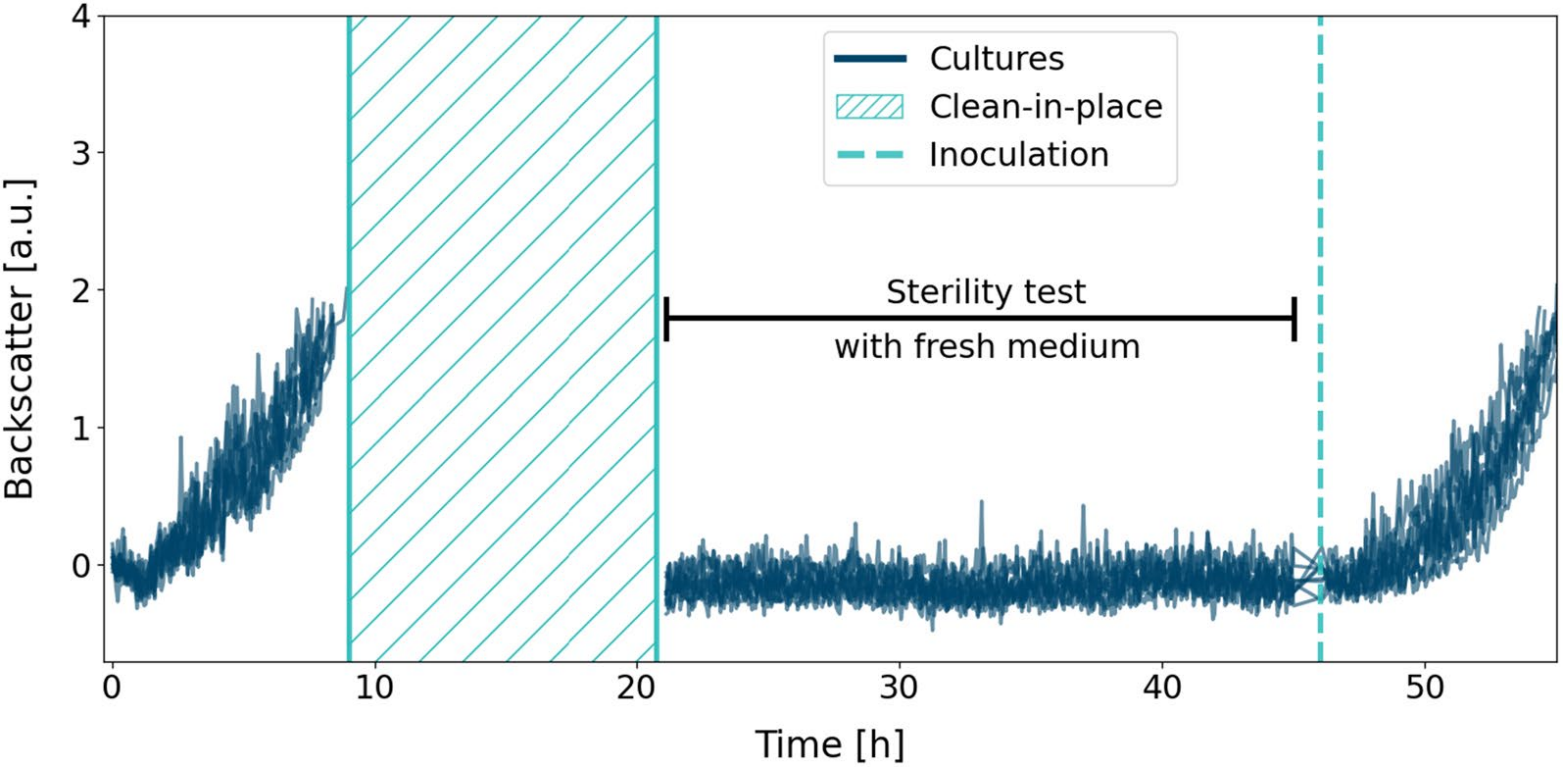
CIP strategy using methanol for disinfectant removal. 12 biological replicates of *C. glutamicum* wild-type were cultivated in a FlowerPlate^®^. After a first batch phase up to a backscatter of 2 a.u., the CIP takes around 11 h. A period of 24 h is used to test for sterile conditions before wells are inoculated from fresh cryo cultures at 46 h process time

A simple way of removing the disinfectant used in this study is evaporation. However, with a vapour pressure of approximately 4 kPa at 20 $^\circ$C [23], evaporation would lead to long process times. Removing the majority of disinfectant by liquid handling and diluting the residual liquid by methanol, which has a vapour pressure of about 13 kPa at the same temperature [24], is thus aiding in shortening the evaporation time. After optimising pipetting velocities and other liquid handling properties for methanol removal (section "Clean-in-place procedure"), 10 h was determined as a sufficient evaporation time (Additional file 1: Fig. S1). The full CIP procedure was tested with *C. glutimacum* as shown in Fig. 4.

First, we performed 12 batch cultivations of *C. glutamicum* wild-type to a backscatter threshold of 2 a.u. In this experiment, the medium in all wells was directly inoculated from cryo cultures. Next, disinfection, washing with methanol and evaporation took place for approximately 11 h as described in section "Clean-in-place procedure" (shaded area). Sterility of the wells after CIP was tested both by incubation of fresh medium for 24 h and by microscopy. Neither microscopy nor backscatter signal indicated contaminants or residual cells from the first cultivation. After the 24-hour period, the wells were inoculated to an OD of 0.2, the same as for the first cultivation. Here, we found that batch times were comparable, further demonstrating a successful CIP. The overall higher noise of the backscatter signal in this particular experiment might be caused by irregularities in the FlowerPlate^®^ or disturbances in the measurement system of the BioLector^®^, since it was not observed in any other experiment under the same process conditions.

Overall, we identified the replacement of disinfectant by methanol and a follow-up evaporation time of 10 h as a suitable CIP strategy. As shown in additional experiments, methanol has the benefit of acting as a second disinfectant since small amounts are toxic to *C. glutamicum* (Additional file 1: Fig. S2). On the other hand, this attribute also helps to identify successful evaporation conditions, as shown in Additional file 1: Fig. S1. During repeated CIP of up to five times, no attrition of adhesive material fusing the FlowerPlate^®^ bottom to the rest of the plate was observed. However, it has to be noted that methanol influences the accuracy and life time of optodes in the FlowerPlates^®^, which was not further investigated in this study. With a first successful demonstration, a remaining point of improvement is the long process time of roughly 11 h, of which 10 h are evaporation. By further optimisation of liquid handling instructions and thus minimising the amount of methanol, the evaporation time could already be reduced to 5 h (Additional file 1: Fig. S1, Table S2). However, more substantial optimisation can be achieved by omitting such evaporation steps completely. This is tackled with the second CIP protocol.

#### CIP with fresh medium for disinfectant removal

**Fig. 5:**
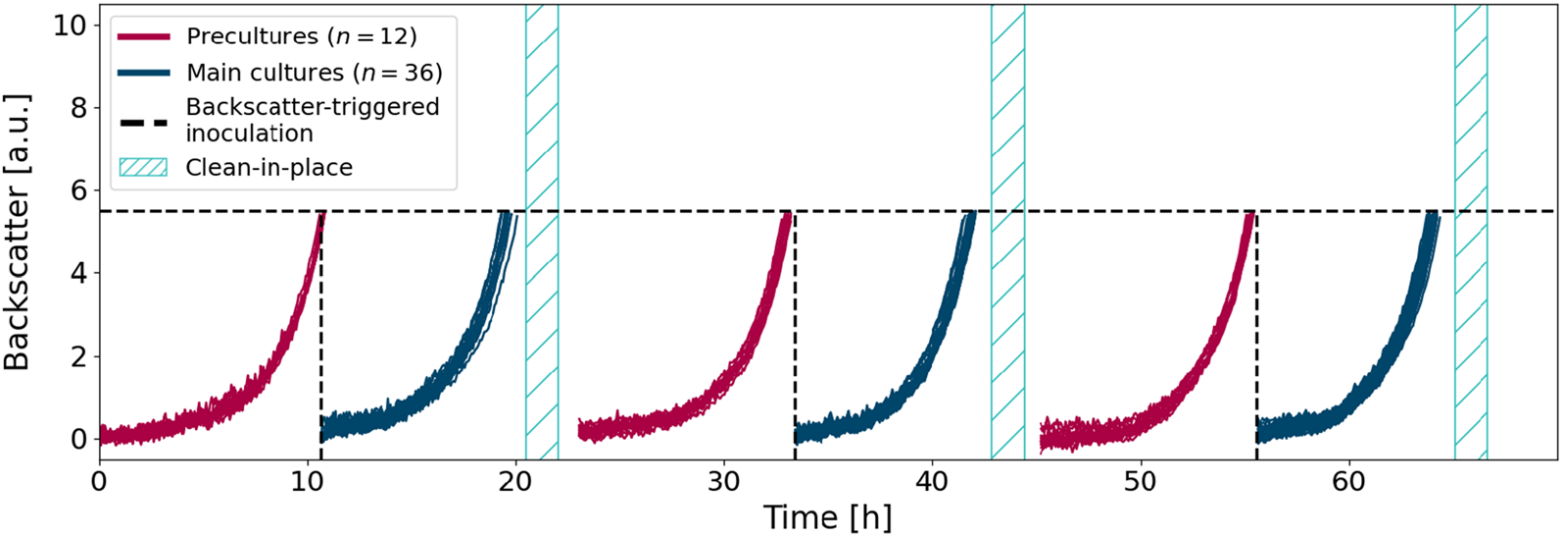
CIP procedure using fresh medium to remove disinfectant. Using the CIP combined with the preculture strategy from section "Step 2: Integrated preculture strategy for high reproducibility", three consecutive cultivations were performed with *C. glutamicum* wild-type. In detail, 12 precultures were cultivated to a backscatter threshold of 5.5 a.u.. Each of those was then used to inoculate three main culture replicates, resulting in 36 replicates overall. These were again harvested upon a threshold of 5.5 a.u.. The shaded area indicates the period of harvesting cells, disinfecting wells and performing CIP, which took about 2 h

A second option to remove residual disinfectant is repeatedly emptying and flushing the wells with sterile medium. Here, it is even more important that no viable cells from the previous cultivation remain in the wells since no additional solvent step is included after disinfection. To determine how many washing steps are needed to remove the disinfectant, we estimated the theoretical amount of residual solvent after sub-optimal liquid handling (Additional file 1: Table S4). Here, already two and three washing steps with sterile medium can reduce the theoretical amount of remaining disinfectant to 0.02% (v/v) and 0.002% (v/v), respectively. Since two and three washing steps resulted in equal growth behaviour to wells without disinfectant treatment (Additional file 1: Fig. S3), the following experiments were performed with a time-efficient 2x-wash CIP. The procedure was tested with *C. glutamicum* as shown in Fig. 5.

For validation of the procedure, it was applied in combination with the preculture strategy described in section "Step 2: Integrated preculture strategy for high reproducibility". With the same layout, 12 precultures (red) were grown in the outermost columns of the FlowerPlate^®^ to 5.5 a.u. (dashed black lines) and then used to inoculate main culture triplicates (blue) in the adjacent inner columns (compare Fig. 3, inlay). At 5.5 a.u. in the main cultures, cells were harvested. Once all 36 main cultures were sampled, liquid removal from the preculture wells and CIP of all 48 wells was triggered. After disinfection, all wells were washed with sterile CGXII medium (shaded area) and freshly inoculated with a new cryo culture.

Growth behaviour was reproducible over the course of the whole procedure and no significant deviation was found for the batch times of three consecutive cultivations (Additional file 1: Table S3). This experiment thus demonstrates that disinfection combined with optimised liquid handling settings (section "Clean-in-place procedure") and medium wash steps is a suitable CIP protocol for *C. glutamicum* for at least three consecutive cultivations. Due to the obsolete evaporation step, process times could be drastically reduced from the 11 h presented in section "CIP strategy with methanol for disinfectant removal" for CIP using methanol to about 2 h for this procedure.

In combination with the automated deep freezer and the preculture strategy, all requirements towards fully autonomous, consecutive cultivations with sampling are fulfilled. In the final result chapter, the methodology was applied in a case study of enzyme secretion.

### Case study: autonomous screening of cutinase-secreting *C. glutamicum* variants

**Fig. 6:**
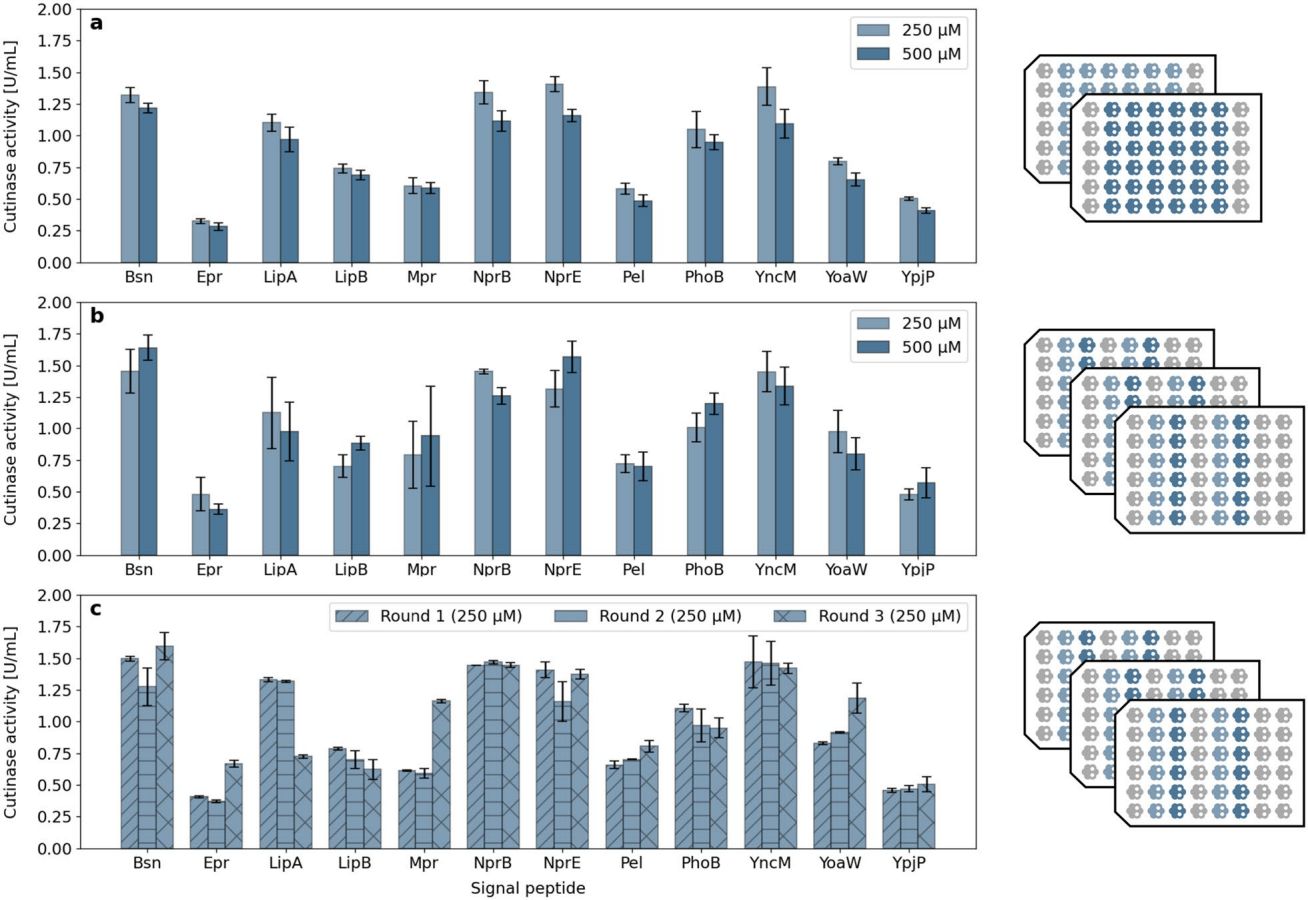
Comparison between different inoculation strategies. **a** Secreted cutinase activity for two IPTG concentrations from biological triplicates placed in the same MTP batch cultivation. **b** Secreted cutinase activity for two IPTG concentrations from biological triplicates which were distributed across different MTP batch cultivations. **c** Results from plot **b** individually shown per batch (round). Batch-to-batch variations between different runs become evident. Error bars show one standard deviation from six replicates (three biological, each with two technical replicates) in **a**, **b** and the difference between technical duplicates in **c**

For application of steps 1 to 3, we chose a case study for autonomous strain library screening. In screening and process optimisation, the benchmark is often defined as the best or a combination of the best performances in historic batch runs, which is referred to as “Golden Batch” [25, 26]. In process monitoring, one task is to detect deviations from this benchmark, e.g. due to unexpected changes in process conditions [27]. When screening a strain library, it is important to ensure that the measured performances are only influenced by the different phenotypes rather than undesired batch-to-batch deviation from the Golden Batch conditions. Here, we thus investigated two different strategies to screen a strain library: In strategy 1, all biological replicates for one condition and strain were placed on the same MTP. In strategy 2, however, these biological replicates were spread over multiple batch experiments (MTPs) to investigate potential batch-to-batch effects (compare Fig. 6, right).

A previously published *C. glutamicum* library for cutinase secretion, mediated by different Sec-type signal peptides from *Bacillus subtilis* [12], was screened in three consecutive batch experiments. For the automated process, WCBs were produced in single-use MTPs as detailed in section "Strain maintenance for cutinase secretion strains". In the automated workflow (section "Screening workflow for *C. glutamicum* cutinase secretion library"), precultures are used to guarantee high cell viability and good reproducibility in the main cultures. The same triplicate design as developed for section "CIP with fresh medium for disinfectant removal" was applied here to inoculate from precultures in their exponential phase. Upon induction with isopropyl-$\beta$-d-thiogalactopyranoside (IPTG), triggered by backscatter threshold in the early exponential phase, cultivation was continued for 4 h before cell harvest and separation of supernatant by centrifugation [12]. Subsequently to sampling of the last cultivation well, enzymatic activity of all samples was detected in an automated cutinase assay (section "Cutinase assay").

As an example for a typical task in bioprocess optimisation, we tested two different IPTG concentrations, i.e., 250 $\upmu$M and 500 $\upmu$M, for induction of the 12 strains. However, the procedure to obtain three biological replicates was different as described above. In the first strategy, all three biological replicates were placed in the same MTP, while in the second, the three biological replicates were distributed to three consecutive MTPs. Thus, in the first strategy (Fig. 6a, right), all main culture replicates are induced with the same IPTG concentration and in the following batch, the concentration is varied for the whole plate. In the second strategy, all main culture replicates per strain are induced with two different IPTG concentration and the biological replicates for the same strain and IPTG concentration are placed independently in the following two batches Fig. 6b, right).

Figure 6 indicates clear differences in the cutinase activity for different signal peptides. When comparing results for strategy 1, where biological replicates are on the same MTP, it can be seen that there are small differences between the inducer concentration, with higher activites for 250 $\upmu$M IPTG (Fig. 6a). The highest activity and hence secretion of active protein can be observed for Bsn, NprB, NprE and YncM. In contrast, Epr, Pel and YpjP show much lower activities. These results are in accordance with previous studies. For example, Hemmerich et al. and Rohe et al. found that signal peptide YpjP had around 50% lower activities compared to NprE [8, 28]. Beside NprE, signal peptides Bsn, NprB and YncM are promising candidates, offering similar performance with respect to enzyme activity after secretion. The latter two were also identified as suitable signal peptides for cutinase in a recent study by Müller et al. [12].

Compared to the deviations between signal peptides, much smaller differences were observed for the two inducer concentrations for the same signal peptide. Overall, the results indicate that 250 $\upmu$M leads to comparable or even higher activities and thus better protein secretion than 500 $\upmu$M, e.g. with approximately 15% higher cutinase activity for NprE and NprB.

However, taking the results from Fig. 6b into account, the interpretation of results changes. This is striking, since the conditions are equal, except for the distribution of the biological replicates to different MTPs. While the same four signal peptides still show highest activity in the assay, a greater differences between 250 $\upmu$M and 500 $\upmu$M can only be seen for NprB. In addition, the standard deviation of replicates increases, which is shown by the error bars. Especially for some variants such as LipA and Mpr, a large error of around 0.6 U mL^−1^ can be seen, originating from batch-to-batch variation of the data.

This is further highlighted in Fig. 6c, in which the results for 250 $\upmu$M are shown for each batch cultivation individually. Interestingly, several signal peptides showed much higher activity in the third batch cultivation, for example Epr, Mpr and YoaW. Strikingly, analysis of the cultivation data of all rounds did not reveal any abnormality in the growth behaviours (Additional file 1: Fig. S8), limiting the batch-to-batch variation to the product formation or detection in the assay. Since the effect cannot be observed for all peptides investigated in the third round, the deviation is unlikely to be caused by erroneous preparation of assay substances. Moreover, positional effects can be excluded since the respective samples have not been placed in close proximity in the assay, but rather distributed over the MTP. This leaves other unfavourable conditions in pre- or main cultures, e.g. the batch of cryo cultures, as a potential reason. As shown in Additional file 1: Fig. S7, the batch-to-batch variation was also observed for an IPTG concentration of 500 $\upmu$M.

With these more detailed results at hand, no inducer concentration is outperforming the other for cutinase expression and secretion in *C. glutamicum*. Moreover, the previously mentioned “Golden Batch” could be observed here, where the first round in strategy 1 (using 250 $\upmu$M) leads to higher activities, but other batches with the same strains and concentration deviate. To avoid false interpretation of results from HT cultivation and enzymatic assays, we thus recommend to spread replicates over multiple batches, thus properly capturing potential batch-to-batch effects. This way, it is guaranteed that screening results can be accounted for by biological rather than technical deviations. In such an experimental design, the same set of IPTG concentrations could be tested over the course of several batches to optimise induction, such as presented in strategy 2.

For this purpose, the developed autonomous workflow for consecutive BioLector^®^ cultivation is well suited, since it allows to embed replicate experiments in short time frames without the need for human interaction. More precisely, the CIP procedure allowed to restore sterile conditions and the highly reproducible growth behaviour indicates that the cryo- and preculture handling is well suited for HT screening setups. In this case study, we could show how the new procedure enables screening of an even larger strain library in shorter times compared to previous studies, thus demonstrating the great capacity for larger screenings in the future.

## Conclusions

In this work, we have presented the necessary steps to conduct autonomous strain phenotyping, an essential part in the Test phase of the DBTL cycle. For this, an automated deep freezer was combined with an automated microbioreactor platform, enabling a greatly increased walk-away time as well as higher utilised capacity of the robotic platform. An integrated preculture strategy allows for high cell fitness and reproducible growth behaviour, which is essential to perform phenotyping experiments under comparable conditions. Two CIP procedures were developed and validated with the goal of overcoming the limitation of manual exchange of MTPs after cultivation. To the best of our knowledge, this is the first time that reconstruction of sterile process conditions was shown for MTPs in automated microbioreactors. This development marks the final step to allow consecutive, autonomous screening experiments, as we demonstrated with a cutinase-secreting strain library.

Overall, our methodology broadens the capacity for phenotyping experiments on automated microbioreactor platforms, allowing to screen larger libraries in even shorter process times. Such acceleration of phenotyping is instrumental to meet the demand of HT strain generation and avoid a bottleneck in screening. While the overall workflow was demonstrated with *C. glutamicum*, the approach can be extended to a larger scope of industrially relevant microorganisms since the methodology is generic. Towards faster iteration of the DBTL cycle, this work shifts the bottleneck from the Test towards the Learn phase, providing large data sets for HT data analysis and decision-making algorithms that are currently developed in the field of Machine Learning.

## Materials and methods

### Software

All analyses and plots presented in this study were performed with recent versions of Python 3.8, matplotlib $\ge$3.5 [29], NumPy $\ge$1.20 [30], pandas $\ge$1.3 [31, 32], SciPy $\ge$1.7 [33] and related packages. For parsing of BioLector^®^ data, the bletl package [34, 35] was applied. Photometric measurements were analysed using the in-house developed retl package (not published). The robotools Python package was used to facilitate multi-step liquid handling instructions on the robotic platform [36].

### Spline analysis for batch time calculcation

The bletl package was used to calculate smoothing splines, an interpolation for the measured backscatter curves. In detail, the function get_crossvalidated_spline was used with the method UnivariateCubicSmoothingSpline to obtain a k-fold cross validated smoothing factor. More details on spline analysis can be found in the bletl software documentation [35].

Since *C. glutamicum* wild-type mostly follows exponential growth kinetics before transitioning into the stationary phase, the first derivative of the spline analysis can be used to detect batch times. More precisely, the maximum of the first spline derivative is a reproducible criterion for batch times, as shown in Additional file 1: Fig S4 for various growth curves.

### Cultivation media and strains

If not indicated otherwise, *C. glutamicum* ATCC 13032 [37] (referred to as wild-type) was used and cultivated at 30 $^\circ$C in CGXI﻿I [7] medium. Cultivation in flasks was done in 500 mL Erlenmeyer shake flasks without baffles at 250 rpm, a filling volume of 50 mL and a shaking diameter of 25 mm. For the application study, a strain library of *C. glutamicum* for secretion of *Fusarium solani* f. sp. *pisi* cutinase was used, which was constructed and provided as cryopreserved cultures as described in [12]. In short, the library consists of pCMEx8 plasmids containing the cutinase gene and 12 different Sec-type signal peptides from *B. subtilis* for protein secretion. An overview of the constructs can be found in the appendix (Additional file 1: Table S5). Media were supplemented with 30 $\upmu$g mL^−1^ kanamycin for cutinase secretion strains.

### Optical density measurements

OD was measured in a UV-1800 spectrophotometer (Shimadzu, Japan) at 600 nm. 0.9% (w/v) NaCl solution was used for sample dilution and as blank.

### Robotic platform for HT cultivation and screening

For automated experiments, a Tecan Freedom EVO^®^ robotic platform was used. The platform includes a liquid handling unit with washable fixed tips, a robotic manipulator arm, an integrated BioLector^®^ Pro microcultivation device (Beckman Coulter), an MTP centrifuge (Rotanta 460 Robotic, Hettich), a cooled carrier for interim sample storage (connected to a Microcool MC 600 circulating chiller, Lauda), a microplate reader (Infinite^®^ M Nano, Tecan) and an MTP shaker (BioShake T-Elm 3000, QInstruments).

Standard cultivation conditions were 30 $^\circ$C, 1400 rpm and $\ge$ 85% relative humidity with measurement of backscatter, pH and DO every 13 min. For plates without optodes, backscatter was measured with a cycle time of 4 min. Experimental procedures were programmed and controlled using the in-house developed DigInBio Control System (Osthege & Hemmerich, manuscript in preparation). Essentially, experimental control scripts programmed in Python implement the experiment logic and procedure, while gateways are used to communicate with the different laboratory devices.

### Integration of automatic deep freezer

For automation of WCB handling, an automatic deep freezer (STX44-DFBT, LiCONiC) was integrated into the robotic platform. To allow flexible usage on different platforms, the deep freezer was placed onto a mobile cart (Additional file 1: Fig. S6). Through a resealable cut-out in the front window of the robotic platform (Additional file 1: Fig. S5) and an in-house developed docking system for accurate alignment of both devices, the freezer can be attached to the robotic system before the start of the experiment; this provides access for the liquid handler and the robotic manipulator arm. For device control, a commercial driver (Tecan, Switzerland) that is compatible with the robotic platform was used. MTPs can either be loaded manually through the front door or from the deck of the robotic platform using the robotic manipulator arm and a smaller, automated door in the back. For minimal thawing, all WCBs were manually loaded to the deep freezer from the front directly after treatment with liquid nitrogen as described in sections "Strain maintenance for cutinase secretion strains" and "Inoculation tests with MTPs".

### Cell viability studies with *C. glutamicum*

For the comparison of storage temperatures (section "Integration of automated deep freezer") in deep freezing, cryo cultures of *C. glutamicum* wild-type were manually prepared in one batch and stored at two different temperatures. First, six shake flask cultures with CGXII medium (10 g L^−1^ glucose) were inoculated to OD 0.1 and incubated for about 15 h as described in section "Cultivation media and strains". The harvest was done manually during the exponential growth phase based on the online monitoring data of an SVR vario system (PreSens Precision Sensing GmbH). After harvesting, 45 mL of each flask were transferred into sterile 50 mL tubes and centrifuged at 4 $^\circ$C and 8000$\times {g}$ for 10 min. Afterwards the supernatant was removed, the cell pellet was resuspended in 0.9% (w/v) sterile sodium chloride solution and mixed in equal parts with 50% (w/v) glycerol solution to reach a final glycerol content of 25% (w/v) and an OD of 30. Aliquots of 500 $\upmu$L were quickly transferred into individual 2 mL cryo vials and frozen with liquid nitrogen. One part of the cryo cultures was stored at −20 $^\circ$C, the other at −80 $^\circ$C.

As a measure for cell viability, batch times from inoculation to the stationary phase were calculated as described in section "Spline analysis for batch time calculcation". To monitor cell viability, a FlowerPlate^®^ without optodes, containing CGXII with 10 g L^−1^ glucose was used. Cultivation was performed in a BioLector^®^ I (Beckman Coulter, Germany) at 30 $^\circ$C, using FlowerPlates without optodes and a gas-permeable sealing foil. Batch times were investigated weekly over the course of six weeks by cultivation and subsequent spline analysis of the growth curves. For each of the storage conditions (−20 $^\circ$C/−80$^\circ$C) and weeks, three different cryo cultures were used, each of those for inoculation of four wells to an OD of 0.1. To avoid deviation due to pipetting errors, a larger volume was inoculated per cryo cultures and distributed to the four wells. Overall, this translates to 12 replicates per storage condition and week, where the three biological replicates provide insight into the variability within cryo cultures stored at the same conditions. The technical replicates, on the other hand, reflect the deviations due to process conditions, sampling and processing as well as analytical methods.

### Inoculation tests with MTPs

For production of WCBs in MTPs, *C. glutamicum* wild-type was cultivated in shake flasks as described in section "Cell viability studies with *C. glutamicum*". Instead of adjusting to a final OD of 30 for Eppendorf Tubes^®^, the cell suspension was adjusted to OD 4 and 8, respectively, and 100 $\upmu$L of solution per well (containing 25% (w/v) glycerol) were transferred to a UV-sterilised flat bottom 96-well MTP. The plates were selaed with self-adhesive aluminium foil, frozen in liquid nitrogen and directly stored in the automated deep freezer at −20 $^\circ$C. Plates were prepared freshly before experiments and used within one week.

To test liquid handling and thawing procedures, a thawing time of 7 min was used for WCBs in MTPs. Whenever substances were pre-heated, they were placed on the MTP shaker on the robotic platform prior to the experiment. Wells with a higher OD of 8 were used in tests with 0.9% (w/v) NaCl solution and 50% (w/v) glycerol. 100 $\upmu$L of the respective solution, pre-heated to 40 $^\circ$C, were added to the wells and mixed three times (100 $\upmu$L aspiration volume), leading to an OD of 4 before inoculation. For the comparison with cryo cultures in Eppendorf Tubes^®^, one cryo culture, produced as described in section "Cell viability studies with *C. glutamicum*", was thawed, diluted from OD 30 to OD 4 using sterile 0.9% (w/v) NaCl solution and manually placed on the robotic deck. For both MTPs and Eppendorf Tubes^®^, 20 $\upmu$L per cryo culture was added to 780 $\upmu$L CGXII medium with 10 g L^−1^ glucose, resulting in OD 0.1 for precultures upon inoculation. Main cultures were inoculated with 20 $\upmu$L of precultures upon a backscatter threshold of 5.5 a.u.

### Clean-in-place procedure

**Fig. 7:**
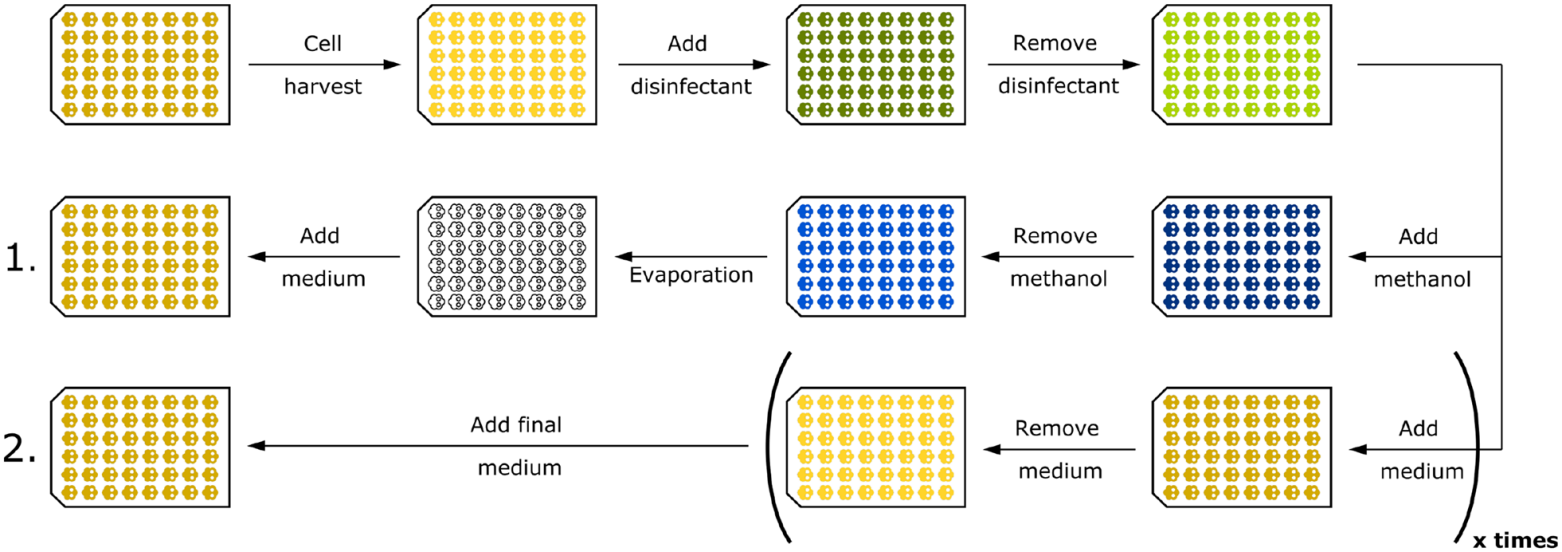
Overview of CIP strategies. In both strategies, disinfection takes place with an ethanol/propan-1-ol mixture which is removed by liquid handling in two steps. In CIP 1, the residual liquid, which was determined to be less than 10 $\upmu$L, is diluted by adding and removing methanol. The residual methanol (< 10 $\upmu$L) is passively removed by evaporation. In CIP 2, the residual disinfectant is diluted by multiple wash steps with fresh CGXII medium

In order to reuse cultivation MTPs in consecutive batches, two CIP procedures were developed. FlowerPlates^®^, sealed with a gas-permeable sealing foil with perforated silicone layer for automation (Beckman Coulter), were used to develop the methods. An overview of the required steps for CIP can be found in Fig. 7.

As a common base between both procedures (Fig. 7, top row), wells are harvested after cultivation, where the majority of liquid is disposed of. For conservative assumptions in calculation, a maximum amount of 100 $\upmu$L was assumed as residual.

If not stated otherwise, the remaining broth in the wells was inactivated using 800 $\upmu$L disinfectant. In accordance with the composition of commercially available disinfectants for microbiology and medical use cases, 23.5 g ethanol and 35.0 g propan-1-ol were mixed and filled up with deionized water to a total weight of 100 g. The disinfectant is incubated under continuous shaking for 20 min at 30 $^\circ$C and 1400 rpm. Afterwards, the remaining liquid is removed using liquid handling settings optimised for solvents. In particular, two removal steps, one with a faster aspiration speed of 250 $\upmu$L s^−1^ and one with 70 $\upmu$L s^−1^ were used. The latter speed was used since slow pipetting speeds are particularly recommended for volatile liquids [38]. All pipetting was operated under shaking to facilitate the cleaning of individual wells in a running process.

As a common goal for both CIP procedures, the remaining disinfectant, which was optimised to be less than 10 $\upmu$L, needed to be removed since it would be toxic for cells in a subsequent cultivation. In the first CIP (middle row), this is achieved by replacing the disinfectant with 400 $\upmu$L methanol, which is removed using the above-described liquid handling instructions for volatile solvents in two steps. The residual methanol, which was measured to be less than 10 $\upmu$L, was passively removed by evaporation under shaking and incubation at 30 $^\circ$C.

In the second CIP procedure (bottom row), the remaining disinfectant is displaced by adding and removing 700 $\upmu$L of sterile CGXII medium. During development, it was tested how many repetitions of medium addition are necessary to remove the residual disinfectant. After the wash steps with sterile medium, 800 $\upmu$L fresh CGXII medium was added to each well for the next cultivation. As a preparation for the final case study, we calculated the theoretical amount of residual cells and disinfectant in the wells (Additional file 1: Table S4). Here, we introduced a modification to the procedure in the disinfection step: Prior to the incubation with 800 $\upmu$L disinfectant for 20 min, 500 $\upmu$L disinfectant were added to the remaining culture broth and removed in two steps with the fast and slower aspiration speed. This step was added since the remaining liquid in the wells could be optimised to be less than 10 $\upmu$L for solvent-like liquids compared to the maximum of 100 $\upmu$L residual liquid that was achieved with the culture broth. In the validation of CIP using medium and in the case study, this additional step was kept.

For both strategies, sterile conditions were validated in a 24-hour incubation of pure medium without inoculum and without antibiotics. In addition, at least three samples were taken at the end of the period and investigated via microscopy. For this, 10 $\upmu$L of the sample were transferred to a microscope slide and covered with a cover slip. The samples were inspected under the 100× lens of an Eclipse TS2R microscope (Nikon, Germany), overall resulting in a 1000× magnification.

Note that the additional wash step prior to disinfection could also be included in the CIP using methanol. Since sterile conditions were already verified for this procedure and the faster method, which is CIP with medium wash steps, was used in the case study, this was not further investigated.

### Strain maintenance for cutinase secretion strains

To create master cell banks (MCBs) for the cutinase secretion strains, 3.5 mL of CGXII medium with 20 g L^−1^ glucose were filled into the wells of two UV-sterilized 24-well plates (Riplate SW, PP, 10 mL, Ritter GmbH). After inoculation to OD 0.4 with 0.05 mL of the respective cryo culture (OD 30), the plates were sealed with a gas-permeable sealing foil. The cultures were incubated for 15.5 h at 30 $^\circ$C, 900 rpm and 3 mm shaking diameter. At the time of harvest, the OD of all cultures was between 30 and 32. 1 mL of chilled 50% (w/v) glycerol was dispensed into all wells of UV-sterilized 24-well plates and mixed with 1 mL of the cell suspension, resulting in a final OD between 15 and 16. The plates were sealed with aluminum foil, frozen using liquid nitrogen, and stored at −80 $^\circ$C.

For screening of the different strains, WCBs were prepared in 96-well MTPs (V-bottom) starting from the MCB. For this purpose, 12 selected strains were cultivated in UV-sterilized 24-well plates with 3 mL CGXII medium (20 g L^−1^ glucose). After inoculation at OD 0.08, the 24-well plates were incubated at 30 $^\circ$C, 900 rpm and 3 mm shaking diameter for 12 h. 50 $\upmu$L of pre-chilled 50% (w/v) glycerol was added to each well of UV-sterilized 96-well plates and mixed with 50 $\upmu$L of the respective culture. The plates were sealed with self-adhesive aluminum foil, frozen with liquid nitrogen and stored at −80 $^\circ$C. In order to avoid multiple thawing of cryopreserved cultures, the MTPs with WCBs were designed for single use.

### Screening workflow for *C. glutamicum* cutinase secretion library

A 96-well MTP with cryopreserved cultures (section "Strain maintenance for cutinase secretion strains") and CGXII medium in a trough were placed on deck of the robotic platform. For precultures, 760 $\upmu$L CGXII was transferred to 12 wells of a FlowerPlate^®^ with pH and DO optodes covered with a gas-permeable sealing foil with perforated silicone layer for automation (Beckman Coulter). This FlowerPlate was either a new plate or freshly prepared by one of the CIP procedures. 40 $\upmu$L of each cryopreserved culture was used to inoculate one of the preculture wells. Remaining cryo culture wells in the MTP not used for preculture were discarded.

For full automation of the process, backscatter-triggered inoculation and induction as well as time-triggered sampling are managed by worklists written with the robotools package and executed by the device control system developed by M. Osthege and J. Hemmerich (Forschungszentrum Jülich).

As soon as a preculture exceeded 5.5 a.u. in backscatter (exponential phase), three main cultures were filled with 780 $\upmu$L CGXII and inoculated with 20 $\upmu$L of the respective preculture. Target protein expression in the main cultures was induced by addition of IPTG to a final concentration of 250 $\upmu$M or 500 $\upmu$M, triggered by a backscatter signal of 3.7 a.u. (early exponential phase). Cells were harvested 4 h after induction and centrifuged for 6 min at 3756$\times g$ and 4 $^\circ$C. Supernatants were stored in a 1 mL deep well plate on a cooling carrier at 4 $^\circ$C until all cultivations were finished and then used for subsequent cutinase activity assays (section "Cutinase assay"). A scheme for the screening workflow with 12 precultures and 36 main cultures can be found in [12].

### Cutinase assay

Activity of cutinase in cultivation supernatant samples was determined spectrophotometrically by degradation of 4-nitrophenyl palmitate as a substrate analogue [39] as described by [12, 18]. In accordance with the standard definition, 1 U is defined in this study as the amount of enzyme that catalyses the conversion of 1 $\upmu$mol of substrate in 1 min. For data analysis, enzymatic activities in U mL^−1^ were calculated in relation to the assay volume with the difference in absorption over time Δ*A*_410_ in a.u. min^−1^, slope of the 4-nitrophenol standard *m*_standard_ in a.u. mM^−1^, obtained from linear regression, and the unitless supernatant dilution factor DF (Eq 1).

1 $$\begin{aligned} \text {EA} = \Delta A_{{410}} \cdot \frac{1}{m_{\mathrm{standard}}} \cdot \text {DF} \end{aligned}$$

## Supplementary Information

**Additional file 1. Table S1:** Batch times of cultures inoculated from cryo cultures stored at −80 °C and −20 °C for *Escherichia coli*. **Table S2:** Batch times of cultures with and without methanol treatment for different evaporation times. **Figure S1:** Comparison of evaporation times. **Figure S2:** Influence of residual methanol on growth behaviour. **Figure S3:** Comparison of CIP with CGXII medium and untreated wells. **Table S3:** Batch times of three consecutive batches with CIP using CGXII medium. **Figure S4:** Exemplary spline analysis for batch time calculation. **Figure S5:** Front window cut-out. **Figure S6:** Mobile cart for freezer. **Figure S7:** Cutinase activity for replicates spread over several batch experiments. **Figure S8:** Backscatter trajectories for Mpr and LipA in strategy with replicates spread over several batches. **Table S4:** Calculation of remaining disinfectant for CIP with CGXII medium for disinfectant removal. **Table S5:** Overview of cutinase secretion strains.

## Data Availability

The data supporting the findings of this study are available within the article and its supplementary materials. Raw data in other formats may be requested from the corresponding author.

## Abbreviations

CIP: Clean-in-place
DBTL: Design-Build-Test-Learn
HT: High-throughput
IPTG: Isopropyl-$\beta$-d-thiogalactopyranoside
MBR: Microbioreactor
MCB: Master cell bank
MTP: Microtiter plate
OD: Optical density
WCB: Working cell bank
