# Supplementary material for: From frozen cell bank to product assay: high-throughput strain characterisation for autonomous Design-Build-Test-Learn cycles
Source: Microb Cell Fact. 2023 Jul 14;22:130. doi: 10.1186/s12934-023-02140-z (PMC10349472; doi:10.1186/s12934-023-02140-z)
Supplement: Supplementary file 1 — Additional file 1. Table S1: Batch times of cultures inoculated from cryo cultures stored at −80 °C and −20 °C for Escherichia coli. Table S2: Batch times of cultures with and without methanol treatment for different evaporation times. Figure S1: Comparison of evaporation times. Figure S2: Influence of residual methanol on growth behaviour. Figure S3: Comparison of CIP with CGXII medium and untreated wells. Table S3: Batch times of three consecutive batches with CIP using CGXII medium. Figure S4: Exemplary spline analysis for batch time calculation. Figure S5: Front window cut-out. Figure S6: Mobile cart for freezer. Figure S7: Cutinase activity for replicates spread over several batch experiments. Figure S8: Backscatter trajectories for Mpr and LipA in strategy with replicates spread over several batches. Table S4: Calculation of remaining disinfectant for CIP with CGXII medium for disinfectant removal. Table S5: Overview of cutinase secretion strains. [file 12934_2023_2140_MOESM1_ESM.pdf]

Supplementary information to article "From Frozen Cell Bank to Product Assay: High-Throughput Strain Characterisation for Autonomous Design-Build-Test-Learn Cycles" (Helleckes et al.)

Table S1: Batch times of cultures inoculated from cryo cultures stored at $-80\,°\mathrm{C}$ and $-20\,°\mathrm{C}$ for *Escherichia coli*. Experiments were conducted as described for *Corynebacterium glutamicum* in Section "Cell viability studies with *C. glutamicum*" of the main manuscript. Instead of CGXII medium, cultivation was performed in M9 medium modified from Sambrook et al., 1989, containing $10\,\mathrm{g\,L^{-1}}$ glucose, $0.001\,\mathrm{g\,L^{-1}}$ biotin and $20.93\,\mathrm{g\,L^{-1}}$ 3-(morpholin-4-yl)propane-1-sulfonic acid (MOPS) buffer. Batch times were investigated weekly over the course of six weeks by cultivation and subsequent spline analysis of the growth curves. For each of the storage conditions and weeks, three different cryo cultures were used, each of those for inoculation of four wells to an optical density (OD) of 0.1. This leads to a number of 12 replicates per storage condition and week. A minimum error in time of $4\,\mathrm{min}$ was assumed since this is the cycle time used for measurements in the BioLector®.

Week	Batch time (-80 °C) [h]	Batch time (-20 °C) [h]
**0**	20.12 ± 0.16	21.28 ± 0.07
**1**	19.70 ± 0.15	21.23 ± 0.25
**2**	20.13 ± 0.11	21.67 ± 0.10
**3**	20.75 ± 0.18	21.97 ± 0.19
**4**	20.19 ± 0.11	22.02 ± 0.41
**5**	20.20 ± 0.12	22.24 ± 0.12
**6**	20.55 ± 0.17	22.70 ± 0.16

Table S2: Batch times of cultures with and without methanol treatment for different evaporation times.
Batch times were calculated from spline analysis as detailed in the main manuscript. The errors reflect the standard deviation across 12 replicates per condition.

Condition	Batch time (OD 0.1) [h]	Batch time (OD 0.2) [h]
**10-hour evaporation**	13.63 ± 0.10	11.94 ± 0.07
**10-hour reference**	13.62 ± 0.08	11.83 ± 0.07
**9-hour evaporation**	13.81 ± 0.15	12.09 ± 0.08
**9-hour reference**	13.73 ± 0.10	12.09 ± 0.08
**5-hour evaporation**	12.60 ± 0.09	11.16 ± 0.07
**5-hour reference**	12.44 ± 0.07	11.05 ± 0.08
**3-hour evaporation**	13.77 ± 0.16	12.11 ± 0.16
**3-hour reference**	12.91 ± 0.17	11.37 ± 0.10

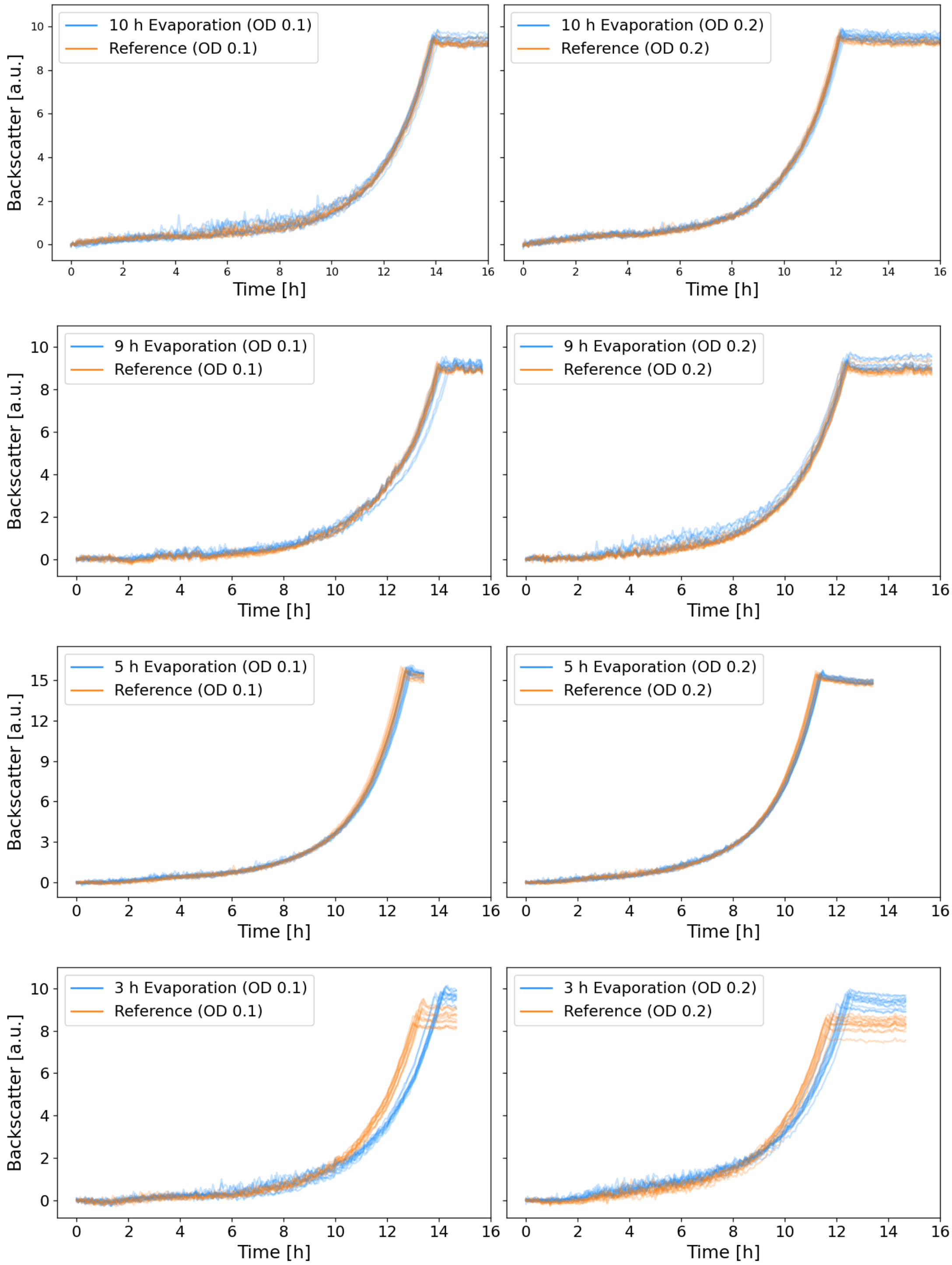

Figure S1: Comparison of evaporation times.
Cultivation was performed at $30\,°\mathrm{C}$, $1\,400\,\mathrm{rpm}$, 85% relative humidity in a BioLector® Pro, using CGXII medium containing $10\,\mathrm{g\,L^{-1}}$ glucose. A FlowerPlate® without optodes was used, sealed with a gas-permeable sealing foil with perforated silicone layer for automation. 24 out of 48 wells were filled with $800\,\mathrm{\mu L}$ methanol, which was subsequently removed in two steps as described in the main manuscript. Evaporation took place under the above-mentioned cultivation conditions. After the respective evaporation time, fresh CGXII medium was filled in all 48 wells and wells were inoculated with *C. glutamicum* wild-type to OD 0.1 or 0.2. Wells without methanol serve as reference.
Evaporation times of $10\,\mathrm{h}$, $9\,\mathrm{h}$ and $5\,\mathrm{h}$ all led to growth times that do not deviate from reference cultures that were not treated with methanol before inoculation. In contrast, $3\,\mathrm{h}$ is not sufficient to evaporate methanol, which can be seen in the slower growth. To guarantee a buffer even for sub-optimal liquid handling in a cultivation process, $10\,\mathrm{h}$ were used for clean-in-place (CIP). Shorter times were not further tested in the complete process since the CIP with medium led to even shorter process times. Batch times were calculated for comparison as shown in Table S2.

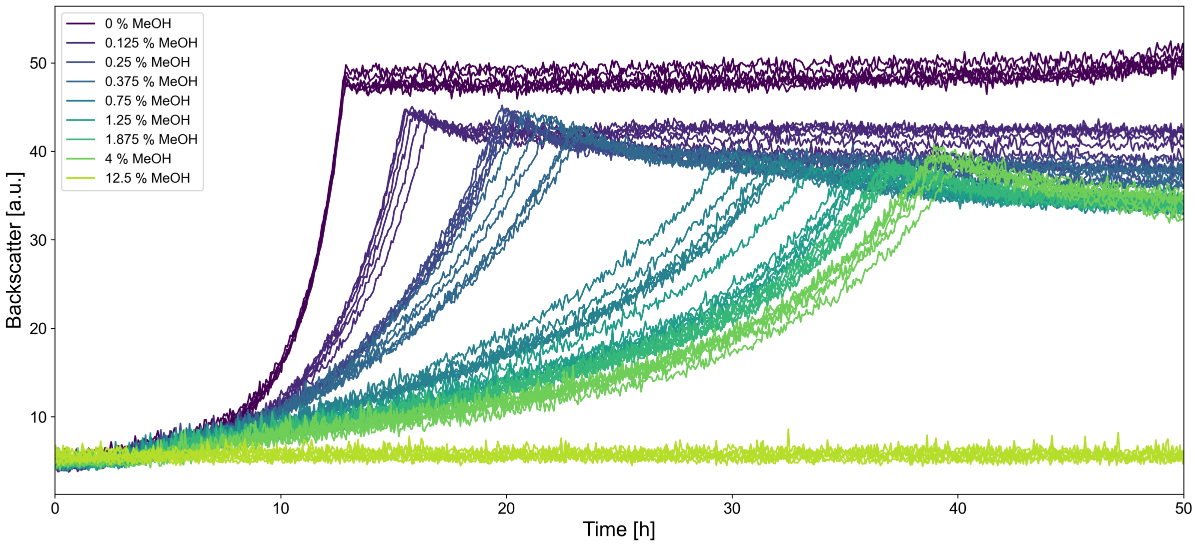

Figure S2: Influence of residual methanol on growth behaviour.
Cultivation of *C. glutamicum* wild-type was performed at $30\,^{\circ}\mathrm{C}$, $1\,400\,\mathrm{rpm}$, 85% relative humidity in a BioLector® I, using CGXII medium containing $10\,\mathrm{g\,L^{-1}}$ glucose and various amounts of methanol. Per concentration, six replicates were cultivated in separated wells of a FlowerPlate® without optodes. The initial OD was 0.1 for all replicates. Even small amounts of 0.125% methanol in CGXII medium, which corresponds to $1\,\mu\mathrm{L}$ in $800\,\mu\mathrm{L}$ cultivation medium, lead to prolonged batch times and a change in signal. This also means that insufficient evaporation times can be easily detected in the backscatter signal, as shown in Fig S1.

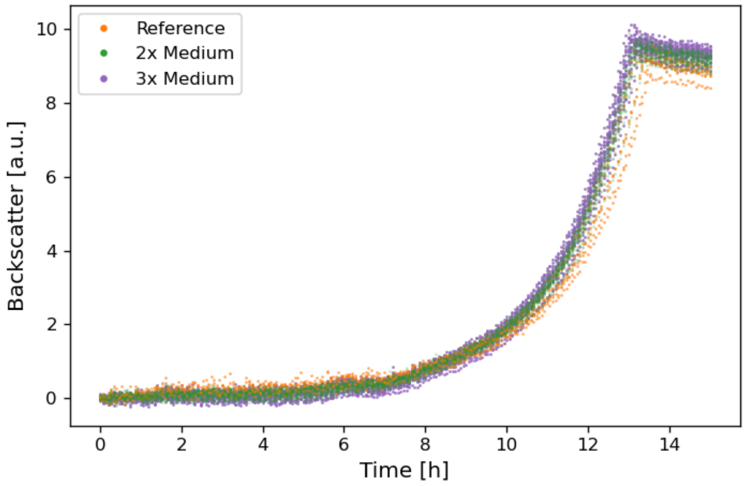

Figure S3: Comparison of CIP with CGXII medium and untreated wells.
Cultivation was performed at $30\,°\mathrm{C}$, $1\,400\,\mathrm{rpm}$, 85% relative humidity in a BioLector® Pro, using CGXII medium containing $10\,\mathrm{g\,L^{-1}}$ glucose. FlowerPlates® sealed with a gas-permeable sealing foil with perforated silicone layer for automation were used. Reference wells were filled with $800\,\mathrm{\mu L}$ CGXII medium inoculated to OD 0.1. For medium wash, medium was filled and removed repetitively as described in the main manuscript.
Untreated wells (orange) and wells with several steps of medium washing (green, purple) show highly comparable growth behaviour. Since the few wells that show slightly delayed growth were those untreated, this effect is more likely to be caused by pipetting errors. A higher amount of residual medium due to accumulation during washing would lead to dilution of the cells at inoculation and thus slower growth, which was not observed here. Due to shorter process times, two instead of three washing steps were thus used in the final CIP procedure.

Table S3: Batch times of three consecutive batches with CIP using CGXII medium.
The calculated batch times are referring to data in Figure 5 of the main manuscript. Batch times were analysed by spline approximation and using the maximum of the first derivative for each replicate as the beginning of the stationary phase. For precultures, 12 biological replicates were analysed. Each of these were used to inoculate three main cultures, resulting in 36 main culture replicates. The second batch is the same data shown in Figure 3 in the main script.

	**Batch time (preculture) [h]**	**Batch time (main culture) [h]**
**Batch 1**	10.40 ± 0.08	8.48 ± 0.12
**Batch 2**	10.12 ± 0.14	8.48 ± 0.10
**Batch 3**	10.14 ± 0.10	8.54 ± 0.41
**Mean**	10.23 ± 0.17	8.50 ± 0.26

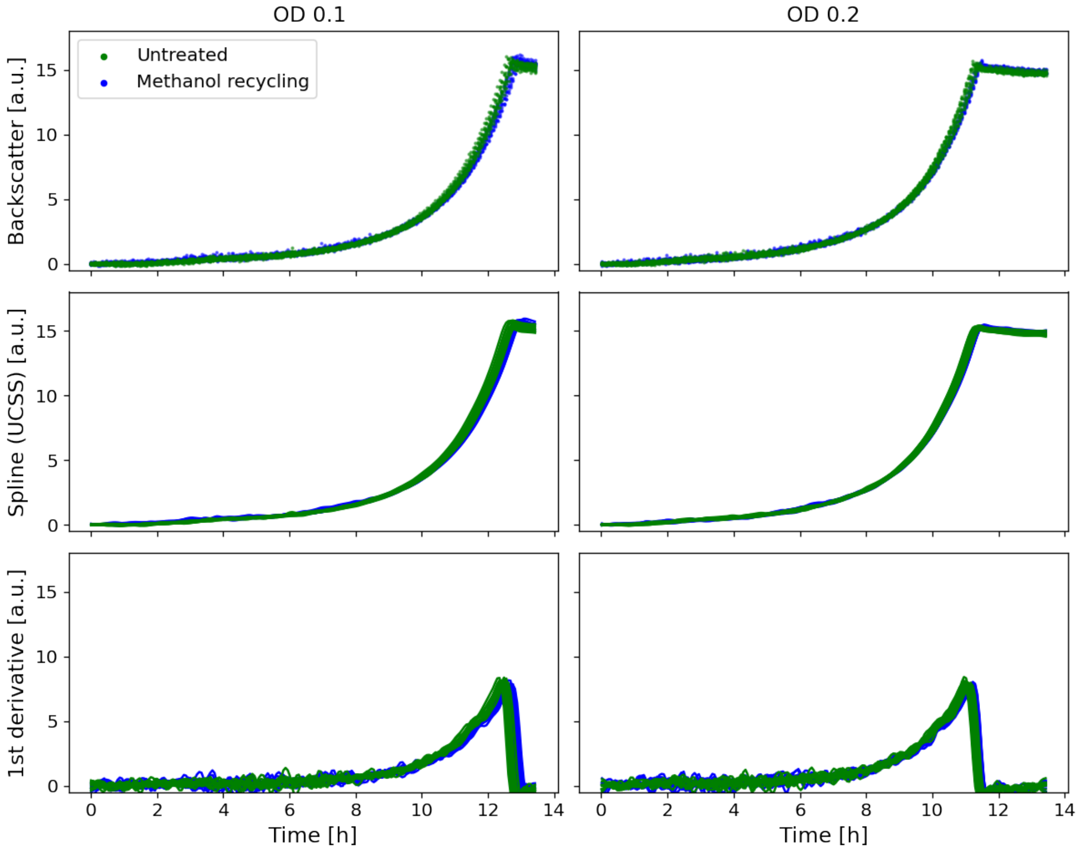

Figure S4: Exemplary spline analysis for batch time calculation.
C. glutamicum wild-type cultures in $800\,\mu L$ CGXII medium were inoculated to the stated OD. In one case, the CIP procedure with methanol was applied to wells before cultivation; in the other case, wells were not treated. Using this example, which shows that the CIP does not affect the batch times, it can be seen how the spline methodology is suited for batch time analysis.
Univariate cubic smoothing splines (UCSS) were calculated with the bletl python package. The first derivative of the splines shows a clear peak at the entry of the stationary phase for *C. glutamicum* wild-type. This maximum was also used to calculate batch times for comparing different cultivation and CIP strategies throughout the paper.

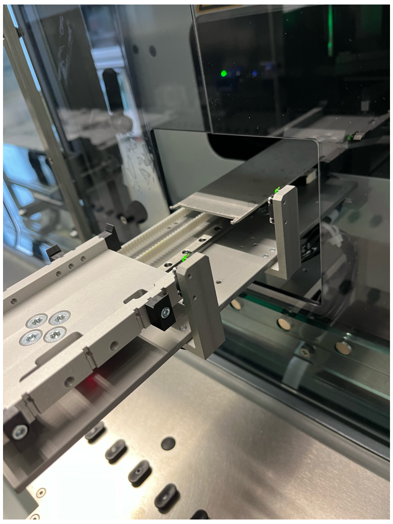

Figure S5: Front window cut-out.
The resealable cut-out in the front window is needed to dock the transfer station of the freezer to the robotic platform. In the picture, the smaller door for automation purposes, located at the back of the freezer, is shown.

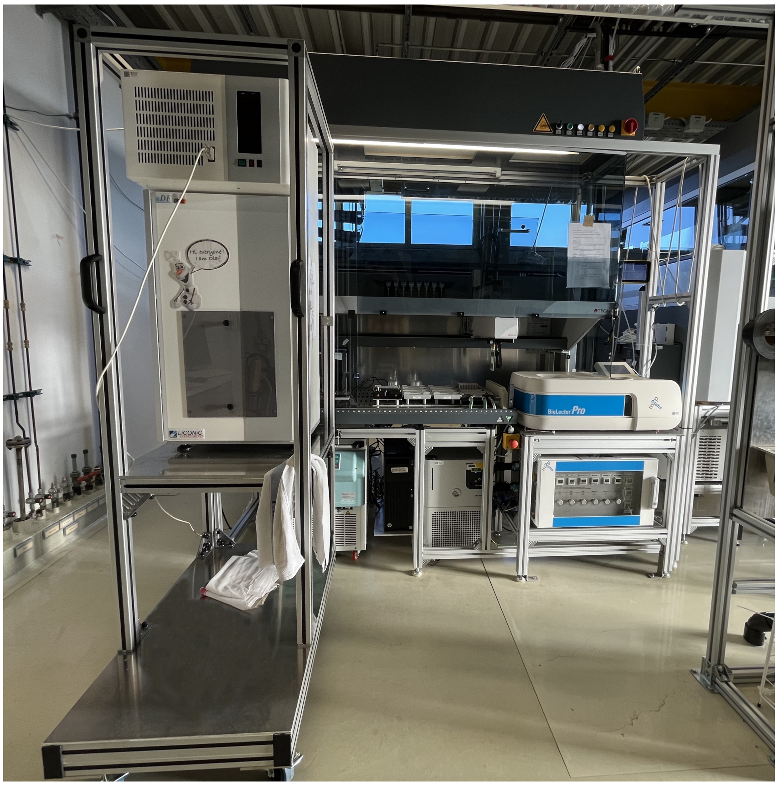

Figure S6: Mobile cart for freezer.
Placing the freezer on a mobile cart allows flexible usage of stored working cell banks (WCBs) on several robotic platforms. Manual loading of microtiter plates (MTPs) is possible through the front door. Automated loading of MTPs is possible via the deck of the robotic platform, using the transfer station shown in Fig S5.

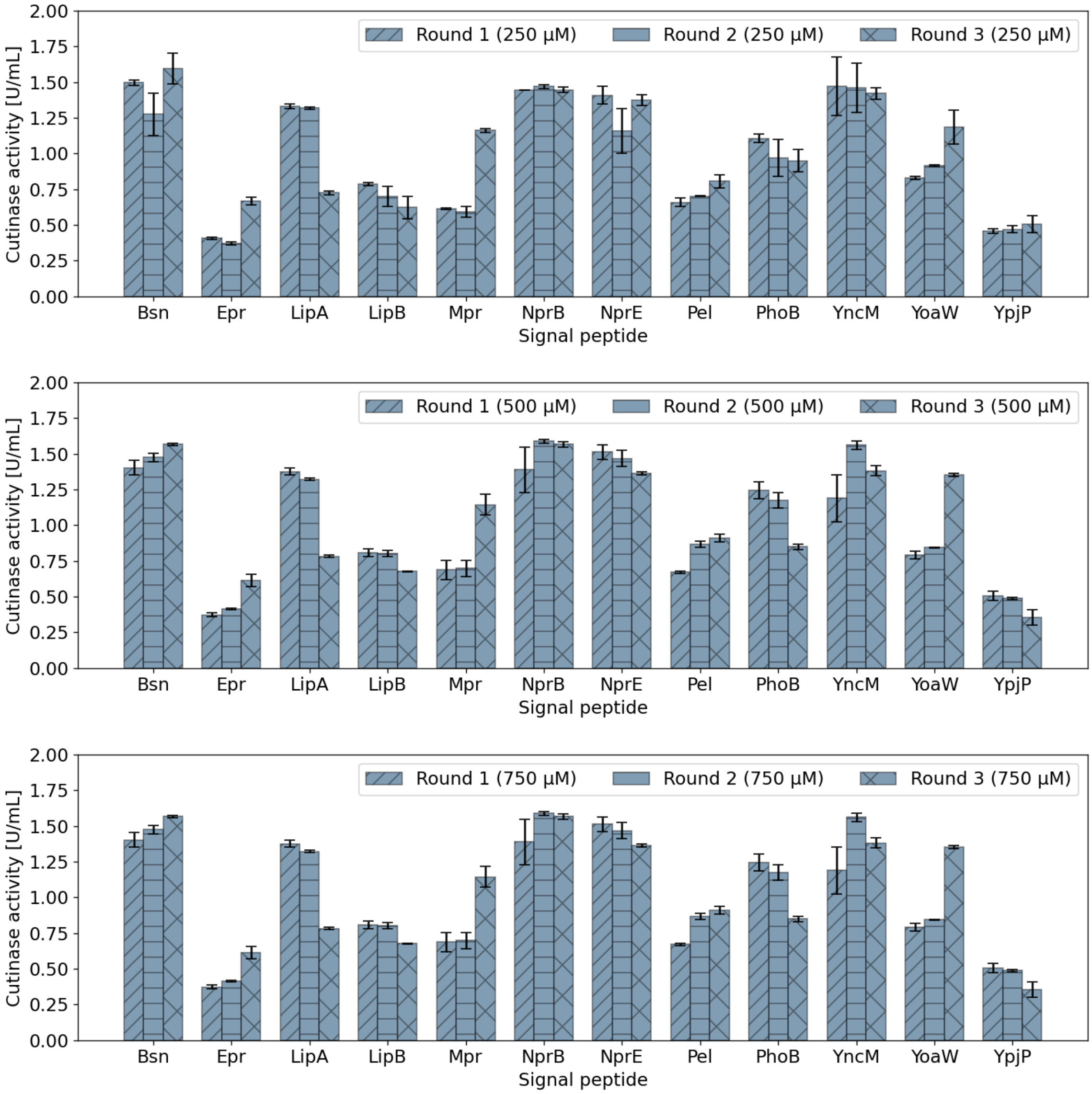

Figure S7: Cutinase activity for replicates spread over several batch experiments.
Three different IPTG concentration between $250\,\mu M$ and $750\,\mu M$ were tested, where all three show very similar results in the activity. This data is complementing the data shown in Figure 6 in the main manuscript.

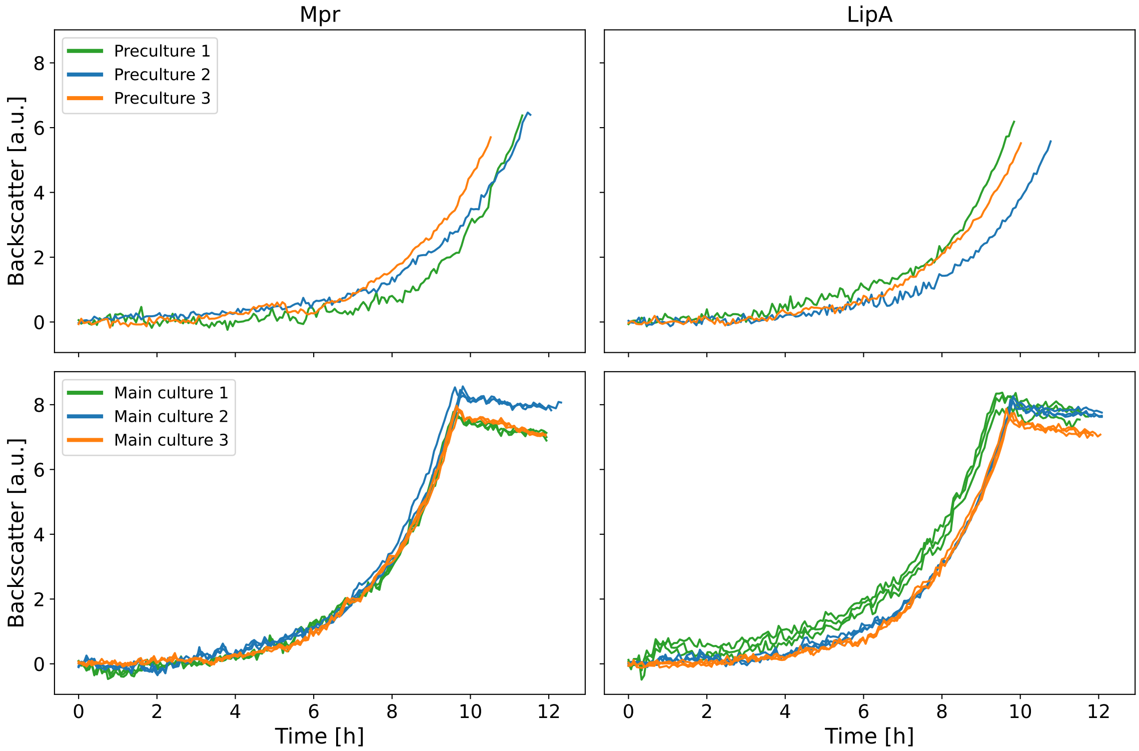

Figure S8: Backscatter trajectories for Mpr and LipA in strategy with replicates spread over several batches. The backscatter data in this figure corresponds to strategy 2 shown in Figure 6 in the main manuscript, meaning the cultures were spread over three different batch cultivations. Although cutinase activities were significantly higher for replicate 3 across several signal peptides, e.g. Mpr, analysis of backscatter did not reveal these effects. In addition, LipA showed a lower activity in the assay for replicate 3, but no evidence for this can be seen in the backscatter of main culture 3. The batch effects might thus be caused either by experimental error in the activity assay or variations in WCBs that cannot be detected in growth, but only influence the amount or activity of cutinase.

Table S4: Calculation of remaining disinfectant for CIP with CGXII medium for disinfectant removal.
The maximum of remaining disinfectant after pipetting was determined to be smaller than $10\,\mu L$ after optimising liquid handling setting. For water-like liquids, it was determined with a maximum of $100\,\mu L$. The final composition is calculated based on these numbers and assuming addition of $800\,\mu L$ medium as a final step after all other liquid handling.

	**Composition during wash steps**		**Remaining in well after CIP**	
**Steps**	Remaining from previous:	Addition of:	Volume after removal:	Final composition (disinfectant/cell density)
Disinfection (1)	max. $100\,\mu L$ cell suspension (OD 20) after harvest	$500\,\mu L$ disinfectant	max. $10\,\mu L$ from disinfection (1)	$8.33\,\mu L$/OD 0.041
Disinfection (2)	max. $10\,\mu L$ from disinfection ($8.33\,\mu L$ disin.; OD 3.33)	$800\,\mu L$ disinfectant	max. $10\,\mu L$ from disinfection (2)	$9.98\,\mu L$/OD 5.1 $\times 10^{-4}$
1x Wash	max. $10\,\mu L$ from disinfection ($9.98\,\mu L$ disin.; OD 4.1 $\times 10^{-2}$)	$700\,\mu L$ medium	max. $100\,\mu L$ from 1x wash	$1.41\,\mu L$/OD 6.4 $\times 10^{-5}$
2x Wash	max. $100\,\mu L$ from 1x wash ($1.41\,\mu L$ disin.; OD 5.8 $\times 10^{-4}$)	$700\,\mu L$ medium	max. $100\,\mu L$ from 2x wash	$0.18\,\mu L$/OD 8.1 $\times 10^{-6}$
3x Wash	max. $100\,\mu L$ from 2x wash ($0.18\,\mu L$ disin.; OD 7.3 $\times 10^{-5}$)	$700\,\mu L$ medium	max. $100\,\mu L$ from 3x wash	$0.02\,\mu L$/OD 1.0 $\times 10^{-6}$

Table S5: Overview of cutinase secretion strains.
All plasmid used in this study were previously published in Müller et al., 2022.

**Plasmid**	**Description**	**Reference**
pCMEx8-[SP]-Cutinase	Based on pPBEx2 (Bakkes et al., 2020), *Escherichia coli*/ *Corynebacterium glutamicum* shuttle vector (Kan^r, pUC18 $oriV_{Ec}$, pBL1 $oriV_{Cg}$, P_{tac}, $lacI^q$), N-terminal *Bacillus subtilis* SP (Bsn, Epr, LipA, LipB, Mpr, NprB, NprE, Pel, PhoB, YncM, YoaW, YpjP), *Fusarium solani* f. sp. *pisi* cutinase, C-terminal GFP-11 tag	Müller et al., 2022

References

Bakkes, P. J., Ramp, P., Bida, A., Dohmen-Olma, D., Bott, M., & Freudl, R. (2020). Improved pEKEx2-derived expression vectors for tightly controlled production of recombinant proteins in Corynebacterium glutamicum. *Plasmid*, *112*, 102540. https://doi.org/10.1016/j.plasmid.2020.102540

Müller, C., Bakkes, P. J., Lenz, P., Waffenschmidt, V., Helleckes, L. M., Jaeger, K.-E., Wiechert, W., Knapp, A., Freudl, R., & Oldiges, M. (2022). Accelerated strain construction and characterization of C. glutamicum protein secretion by laboratory automation. *Applied Microbiology and Biotechnology*, *106*(12), 4481–4497. https://doi.org/10.1007/s00253-022-12017-7

Sambrook, J., Fritsch, E. F., & Maniatis, T. (1989). Molecular cloning: A laboratory manual. [Publisher: Cold Spring Harbor Laboratory Press]. *Molecular cloning: a laboratory manual.*, (Ed. 2). Retrieved June 7, 2023, from https://www.cabdirect.org/cabdirect/abstract/19901616061
